# Ultra-processed food consumption and atherosclerosis risk: a prospective cohort study of UK Biobank

**DOI:** 10.3389/fnut.2026.1925613

**Published:** 2026-09-10

**Authors:** Yixin Zhao, Yuguang Li, Xiangliang Liu, Yuguang Zhao

**Affiliations:** Cancer Center, The First Hospital of Jilin University, Changchun, China

**Keywords:** atherosclerosis, cardiovascular disease, cohort study, metabolomics, proteomics, UK biobank

## Abstract

**Background:**

Ultra-processed foods (UPFs) have become dominant in modern diets, yet their relationship with atherosclerosis—the primary pathological basis of cardiovascular disease—remains inadequately characterized. We investigated the association between UPF consumption and incident atherosclerosis risk and explored underlying metabolomic and proteomic molecular signatures.

**Methods:**

We conducted a prospective analysis of 133,797 UK Biobank participants free of atherosclerosis at baseline. Dietary intake was assessed using 24-h dietary recalls, and UPF consumption was categorized according to the NOVA classification system. Incident atherosclerosis cases were identified through hospital admission records and death registries. Cox proportional hazards models were used to estimate hazard ratios (HRs) and 95% confidence intervals (CIs). In subset analyses, we performed nuclear magnetic resonance metabolomic profiling and plasma proteomic analysis using the Olink platform to identify molecular signatures associated with UPF-related atherosclerosis risk.

**Results:**

During a median follow-up of 10.93 years (interquartile range, 10.83–11.40 years), 654 incident cases of atherosclerosis were documented. After multivariable adjustment, participants in the highest quartile (Q4) of servings/day showed an elevated risk (HR 1.40, 95% CI 1.12–1.75; *p* = 0.003) compared to the lowest quartile (Q1). Similarly, participants in Q4 of UPF consumption (g/day) had a 29% increased risk of atherosclerosis compared to those in Q1 (HR 1.29, 95% CI 1.03–1.60; *p* = 0.024). Restricted cubic spline analysis revealed higher UPF intake was associated with higher risk. Sparse LASSO identified UPF-related signatures comprising 30 metabolites and 25 proteins, predominantly involving lipoprotein, energy-metabolic, inflammatory, and endocrine pathways. Several biomarkers remained associated with incident atherosclerosis after multivariable adjustment and multiple-testing correction.

**Conclusion:**

Higher UPF consumption is associated with increased atherosclerosis risk. Distinct metabolomic and proteomic signatures characterized by systemic inflammation, lipid dysregulation, and enzymatic alterations may reflect potential pathways. These findings support dietary recommendations emphasizing minimally processed foods for cardiovascular disease prevention and highlight candidate molecular correlates requiring independent validation.

## 1 Introduction

Atherosclerosis, characterized by the accumulation of lipids, inflammatory cells, and fibrous elements within arterial walls, remains the primary pathophysiological basis for cardiovascular disease (CVD), the leading cause of mortality worldwide (1, 2). Despite substantial advances in pharmacological interventions and surgical techniques, the global burden of atherosclerotic cardiovascular disease continues to escalate, accounting for approximately 17.9 million deaths annually (3). This persistent challenge underscores the critical need to identify modifiable risk factors, particularly dietary patterns, that may influence disease onset and progression.

Over the past two decades, the Western diet has undergone a fundamental transformation, with ultra-processed foods (UPFs) now constituting a substantial and increasing proportion of daily caloric intake (4, 5). According to the NOVA food classification system, UPFs are defined as industrial formulations typically containing five or more ingredients, including substances rarely used in culinary preparations such as hydrogenated oils, modified starches, protein isolates, and various cosmetic additives (6). Evidence from high-income countries indicates that UPFs account for 50%–60% of total energy intake in the United States and United Kingdom, with similarly concerning trends emerging in middle-income nations (7, 8). This dietary shift represents a profound departure from traditional whole-food-based diets and warrants rigorous investigation of its health implications.

Emerging epidemiological evidence has linked high UPF consumption to elevated risks of obesity, type 2 diabetes, hypertension, and all-cause mortality (9–12). Mechanistically, UPFs are characterized by unfavorable nutritional profiles, including high energy density, elevated sodium and sugar content, excessive saturated and trans fatty acids, and diminished fiber and micronutrient levels (13, 14). Beyond nutritional inadequacy, the industrial processing itself may introduce or concentrate potentially harmful compounds, such as advanced glycation end products, acrylamide, and endocrine-disrupting chemicals from packaging materials (15, 16). These constituents could plausibly promote atherosclerotic processes through multiple pathways, including oxidative stress, chronic low-grade inflammation, endothelial dysfunction, and dysregulation of lipid metabolism (17, 18).

Despite mounting concern, the specific relationship between UPF consumption and atherosclerosis risk remains inadequately characterized. Previous studies have primarily examined composite cardiovascular events, whereas evidence regarding clinically recorded atherosclerosis coded as ICD-10 I70 remains limited (19, 20). This administrative endpoint reflects clinically recognized disease rather than the complete biological process or subclinical plaque burden. Furthermore, the potential molecular pathways linking UPF intake to atherosclerotic disease are poorly understood. Recent advances in high-throughput omics technologies offer unprecedented opportunities to elucidate these pathways. Metabolomics enables comprehensive profiling of small-molecule metabolites that reflect the integrated effects of diet, metabolism, and disease processes (21, 22), while proteomics provides insights into functional alterations in circulating proteins involved in inflammation, thrombosis, and vascular remodeling (23, 24). To date, no large-scale prospective cohort study has systematically integrated dietary assessment with multi-omics profiling to investigate the UPF-atherosclerosis relationship and its underlying molecular signatures.

To address these knowledge gaps, we leveraged data from the UK Biobank, a large-scale prospective cohort with comprehensive dietary, clinical, and biomarker information. Our objectives were threefold: (1) to quantify the prospective association between UPF consumption and incident atherosclerosis risk; (2) to explore potential effect modification by demographic, lifestyle, and metabolic factors; and (3) to identify and characterize specific metabolomic and proteomic signatures that may reflect potential pathways between UPF intake and atherosclerotic disease. By integrating epidemiological analysis with systems biology approaches, this investigation seeks to provide robust evidence for dietary guidelines and to illuminate potential hypotheses for future mechanistic investigation for atherosclerosis prevention.

## 2 Methods

### 2.1 Study design and participants

**Figure 1:**
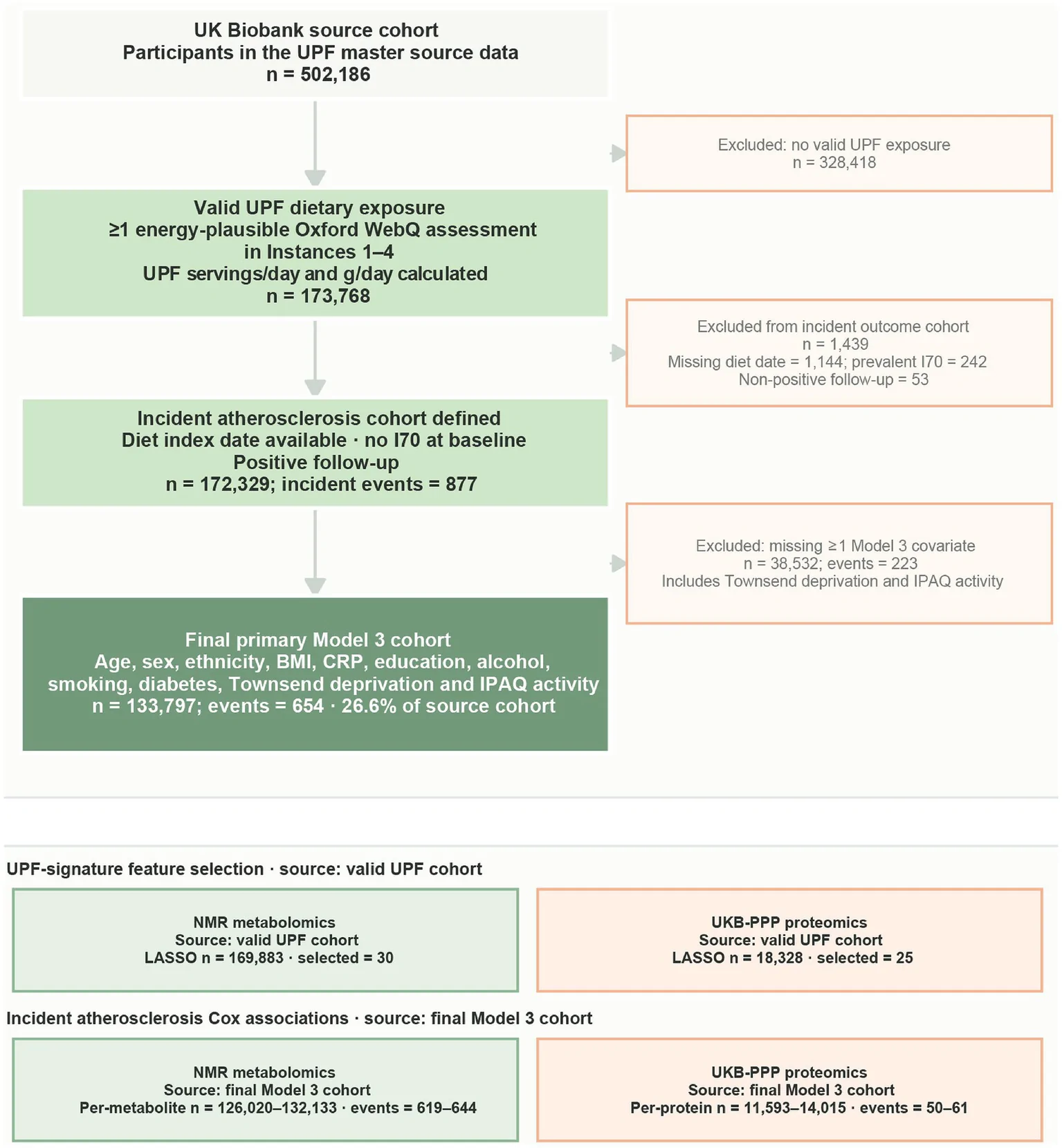
Flowchart of participants from UK Biobank in this study.

This study used data from the UK Biobank (people living in communities in England, Scotland or Wales). Participants completed touchscreen questionnaires, physical measures, and provided biological samples. All UK Biobank participants provided informed consent, obtained electronically and documented by the UK Biobank, for participation and linkage to health records. No additional participant consent was required for this secondary analysis of deidentified UK Biobank data. The study first calculated individual UPF exposure using repeated Oxford WebQ dietary assessments. The date of the last valid dietary assessment was defined as time zero for dietary follow-up. Follow-up accrued from this date until the first recorded diagnosis of atherosclerosis, death, loss to follow-up, or the end of record availability, whichever occurred first (March 31, 2023 for England, August 31, 2022 for Scotland, and May 31, 2022 for Wales). In this study, we excluded 328,418 individuals who lacked valid UPF exposure data. Then, 1,439 individuals who lacked diet data, with prevalent atherosclerosis, or non-positive follow-up were excluded. The individuals without data for core covariables (*n* = 38,532) were also excluded. Finally, the analysis included 133,797 participants. This study followed the Strengthening the Reporting of Observational Studies in Epidemiology (STROBE) reporting guideline. The inclusion and exclusion flow chart for this study is shown in Figure 1.

### 2.2 Ascertainment of clinically coded atherosclerosis

Incident clinically coded atherosclerosis was defined as the first occurrence of the International Classification of Diseases, Tenth Revision (ICD-10) code I70 recorded in linked hospital inpatient or mortality records after the last valid Oxford WebQ assessment. We used UK Biobank field 131,380 and verified its underlying data sources according to the UK Biobank first-occurrence mapping. The date of first I70 reported was obtained from UK Biobank fields 131,380. Participants with prevalent I70 before last valid Oxford WebQ dietary assessment were excluded. Data sources and participant numbers at each screening stage for atherosclerosis in the UK Biobank was shown in Supplementary Table S1.

### 2.3 Dietary assessment and exposure definition

Dietary data were collected using the Oxford WebQ, a web-based 24-h dietary recall tool designed for large-scale epidemiological studies (25). This instrument records the specific types and quantities of approximately 200 common food and beverage items, allowing for the estimation of total energy and nutrient profiles based on intake from the preceding 24 h. The assessment was administered between 2011 and 2012, with the majority of participants completing the questionnaire one to four times at intervals of 3–4 months. Ultra-processed foods (UPFs) were identified using the NOVA classification system, which categorizes foods into four groups based on processing extent and purpose (26, 27). For each valid dietary recall, daily servings, grams, and energy intake were calculated for each food item using standard portion sizes and food energy-density values. For participants with multiple valid WebQ assessments, item-specific daily intakes were averaged across all available recalls before summation. The averaged intakes of UPF items were then summed to derive participant-level UPF intake, expressed as servings/day and grams/day; participants with one valid assessment contributed the value from that assessment. To preserve temporal ordering, follow-up began on the date of the last dietary assessment included in the participant-specific average. Individuals with implausible energy intakes (>4,200 or <800 kcal/day for men; >3,500 or <500 kcal/day for women) were excluded.

### 2.4 Metabolic and proteomic profiling

Metabolic biomarkers were measured from randomly selected EDTA plasma samples (aliquot 3) using a high-throughput NMR-based metabolic biomarker profiling platform developed by Nightingale Health Ltd. The platform quantified 251 metabolic biomarkers, encompassing 170 directly measured metabolites and 81 ratios of them. All samples were collected under standardized protocols and processed centrally to ensure consistency and quality control. The UK Biobank Pharma Proteomics Project (UKB-PPP) is a collaboration between the UK Biobank (UKB) and 13 biopharmaceutical companies characterising the plasma proteomic profiles of 54,306 UKB participants. Prior to all analyses, all metabolite and protein values underwent logarithmic transformation followed by standardization into z-scores.

### 2.5 Assessment of covariables

Potential confounders included age, sex, self-reported race and ethnicity [Asian, Black, Mixed, White, or other race or ethnicity (specific data for the category “other” were unavailable)], education, Townsend deprivation index, smoking status, alcohol use, International Physical Activity Questionnaire (IPAQ) physical activity, body mass index (BMI), C-reactive protein (CRP), doctor-diagnosed diabetes, Dietary Approaches to Stop Hypertension (DASH) diet score, total energy intake, systolic blood pressure, LDL-C and the use of lipid-lowering or antihypertensive medications.

### 2.6 Statistical analysis

Continuous variables are reported as mean (SD) or median (IQR) and categorical variables as frequency (%). Baseline characteristics were compared by one-way ANOVA and Kruskal–Wallis tests. Sequential Cox models were fitted to separate confounding control from adjustment for potential intermediates: Model 1 was unadjusted; Model 2 included age, sex, ethnicity, education, Townsend deprivation index, smoking, alcohol use, and IPAQ physical activity; Model 3 further added BMI, CRP, and doctor-diagnosed diabetes. Model 4 further adjusted for the DASH diet score, whereas Model 5 additionally accounted for total energy intake. Model 6 included systolic blood pressure and LDL-C as additional covariates. Finally, Model 7 also considered the use of lipid-lowering or antihypertensive medications. Nonlinearity indicated by Martingale residuals was assessed by restricted cubic splines with knots at the 5th, 35th, 65th, and 95th percentiles of UPF intake, using the exposure-specific median as reference (HR = 1.00); estimates span the 1st–99th percentiles. Multiplicative interaction was tested by likelihood ratio tests and additive interaction by the relative excess risk due to interaction. Cox regression, restricted cubic spline, subgroup, sensitivity, and missing-data analyses were performed in R version 4.6.0. All tests were two-sided, and *p* < 0.05 was considered statistically significant unless otherwise specified.

### 2.7 Missing-data analyses

Missing data for Model 3 covariates were handled using multiple imputation by chained equations (MICE) under the missing-at-random assumption. Twenty imputed datasets were generated, with 10 iterations per imputation chain, using a random seed of 20,260,811 and eight parallel workers. Binary variables were imputed using binary logistic regression; educational attainment, alcohol consumption status, and International Physical Activity Questionnaire (IPAQ) category were imputed using multinomial logistic regression; and the Townsend index was imputed using predictive mean matching. Age at dietary assessment and sex were complete and therefore not imputed.

To ensure direct comparability with the primary complete-case analysis, the UPF quartile cut points, quartile-specific median values used for trend tests, and standardization parameters for estimating associations per 1-SD increment were fixed to those prespecified in the locked primary complete-case analysis and were not re-estimated after imputation. Cox proportional hazards models were fitted separately in each imputed dataset, with tied event times handled using the Efron method. Regression coefficients, standard errors, 95% confidence intervals, and *p* values were subsequently pooled according to Rubin’s rules. Tests for trend were conducted by modeling the fixed median UPF intake value within each quartile as a continuous variable. All analyses were performed using R, with the mice package (version 3.19.0) and the survival package (version 3.8.6).

### 2.8 Omics preprocessing and penalized feature selection

Baseline Nightingale NMR metabolites and UKB-PPP protein measurements were analysed as candidate molecular predictors of habitual UPF intake. Habitual UPF intake was calculated as described in Methods Section 2.3. Because the distribution of UPF intake was right-skewed, log1p (UPF g/day) was used as the continuous response variable in the penalized regression. Incident atherosclerosis was not used as the penalized-regression outcome but was examined separately in subsequent Cox regression analyses.

The NMR analysis included 169,883 participants with at least one measured baseline NMR metabolite. A total of 3,885 participants with no measured NMR metabolite were excluded. The proteomic analysis included 18,328 participants overlapping with UKB-PPP, all of whom had at least one measured retained protein. Features with more than 20% missingness or zero or invalid variance were excluded. Proteins were additionally required to have at least 1,000 non-missing observations, resulting in 2,907 retained proteins. The UPF response variable was not imputed. Remaining missing omics values were median-imputed, and the features were centred and scaled to unit variance.

For the NMR analysis, Elastic Net models evaluated 60 alpha values and l1_ratio values of 0.10, 0.50, 0.90, 0.95, and 1.00. Five-fold outer cross-validation was used for out-of-fold performance assessment, with five-fold cross-validation within each outer training set for hyperparameter selection. The full-cohort model was subsequently fitted using 10-fold cross-validation. Because the selected l1_ratio was 1.00, the final sparse model corresponded to LASSO. An alpha of 0.002307 was selected from within the one-standard-error range to obtain a sparse signature. Stability selection comprised 30 repeated 80% subsamples without replacement. Metabolites were retained when their selection frequency was ≥80%, their sign consistency when selected was ≥90%, and their full-cohort coefficient was non-zero.

For the proteomic analysis, LASSO (l1_ratio = 1.00) was fitted using 10-fold cross-validation over 36 alpha values ranging from alpha_max to 0.12 × alpha_max. Candidate penalties producing 25–100 non-zero coefficients were evaluated, and the alpha producing a stable feature count closest to 30 was selected. The selected alpha was 0.0289395, which was stronger than the conventional one-standard-error range and yielded 25 stable proteins. Stability was evaluated using the same 30 repeated 80% subsamples and selection thresholds used for the NMR analysis. For final full-cohort model fitting and stability selection, imputation and standardization parameters were estimated using the complete analytic cohort and held fixed during subsampling. For out-of-fold assessments, imputation and standardization were re-estimated within each training fold and applied to the corresponding held-out fold. No separate random test set was reserved. The fixed-alpha five-fold out-of-fold assessments of the final sparse signatures were considered descriptive because alpha selection used the complete dataset. Analyses were performed using Python 3.13.9 and scikit-learn 1.8.0 with fixed random seeds of 20,260,718 for NMR and 20,260,719 for proteomics.

## 3 Results

### 3.1 Participant characteristics

Baseline characteristics of the 133,797 participants according to quartiles of UPF intake (g/day) are shown in Table 1. Median UPF intake increased progressively from 239.0 g/day in Q1 to 1,147.0 g/day in Q4. Mean age was 58.6 years, and women comprised 53.3% of the overall cohort. Across increasing UPF quartiles, the proportions of men, participants with obesity, lower educational attainment, higher C-reactive protein concentrations, and current smoking generally increased. Participants in higher UPF quartiles were more likely to report low physical activity. Doctor-diagnosed diabetes was most prevalent in Q4. All characteristics differed significantly across quartiles (*p* < 0.001). Supplementary Table S2 presents the characteristics of participants across quartiles of UPF intake, quantified in servings per day. The trends of the variables were consistent with those observed in Table 1.

**Table 1:** Baseline characteristics of the participants.

Characteristics	Q1	Q2	Q3	Q4	*p*
	(*n* = 33,454)	(*n* = 33,447)	(*n* = 33,474)	(*n* = 33,422)	
UPF intake, g/day (median [IQR])	239.0 [169.8–295.4]	457.0 [402.7–514.0]	711.2 [639.0–792.2]	1147.0 [996.0–1379.7]	
Age at diet baseline, years, Mean (SD)	58.7 (7.7)	59.0 (7.8)	59.0 (7.9)	57.9 (8.1)	<0.001
Race or ethnicity (%)					<0.001
White	32,012 (95.7)	32,390 (96.8)	32,611 (97.4)	32,699 (97.8)	
Non-White	1,442 (4.3)	1,057 (3.2)	863 (2.6)	723 (2.2)	
Education (%)					<0.001
College	18,729 (56.0)	16,712 (50.0)	14,927 (44.6)	13,112 (39.2)	
High school	13,211 (39.5)	14,857 (44.4)	16,339 (48.8)	17,783 (53.2)	
Less than high school	1,514 (4.5)	1,878 (5.6)	2,208 (6.6)	2,527 (7.6)	
Sex (%)					<0.001
Female	20,221 (60.4)	18,372 (54.9)	17,079 (51.0)	15,612 (46.7)	
Male	13,233 (39.6)	15,075 (45.1)	16,395 (49.0)	17,810 (53.3)	
Alcohol drinking status (%)					<0.001
Never	873 (2.6)	866 (2.6)	879 (2.6)	952 (2.8)	
Previous	897 (2.7)	826 (2.5)	791 (2.4)	1,159 (3.5)	
Current	31,684 (94.7)	31,755 (94.9)	31,804 (95.0)	31,311 (93.7)	
Ever smoked (%)					<0.001
No	13,117 (39.2)	13,625 (40.7)	13,471 (40.2)	12,772 (38.2)	
Yes	20,337 (60.8)	19,822 (59.3)	20,003 (59.8)	20,650 (61.8)	
CRP group (%)					<0.001
Below median	18,369 (54.9)	17,737 (53.0)	16,794 (50.2)	15,354 (45.9)	
At or above median	15,085 (45.1)	15,710 (47.0)	16,680 (49.8)	18,068 (54.1)	
BMI category, kg/m^2^, (%)					<0.001
<30	28,611 (85.5)	28,027 (83.8)	26,862 (80.2)	24,446 (73.1)	
≥30	4,843 (14.5)	5,420 (16.2)	6,612 (19.8)	8,976 (26.9)	
Doctor-diagnosed diabetes (%)					<0.001
No	32,506 (97.2)	32,418 (96.9)	32,248 (96.3)	31,676 (94.8)	
Yes	948 (2.8)	1,029 (3.1)	1,226 (3.7)	1,746 (5.2)	
Townsend deprivation index, Mean (SD)	−1.35 (2.99)	−1.75 (2.80)	−1.90 (2.74)	−1.79 (2.78)	<0.001
Physical activity (IPAQ) (%)					<0.001
Low	5,646 (16.9)	5,821 (17.4)	6,149 (18.4)	6,754 (20.2)	
Moderate	14,358 (42.9)	14,651 (43.8)	14,442 (43.1)	13,858 (41.5)	
High	13,450 (40.2)	12,975 (38.8)	12,883 (38.5)	12,810 (38.3)	

Characteristics of participants are presented as mean (SD) for continuous variables, and frequency (%) for categorical variables. UPF, ultra-processed food; CRP, C-reactive protein; BMI, body mass index.

UPF: Grams/day.

### 3.2 Association between UPF consumption and risk of atherosclerosis

**Figure 2:**
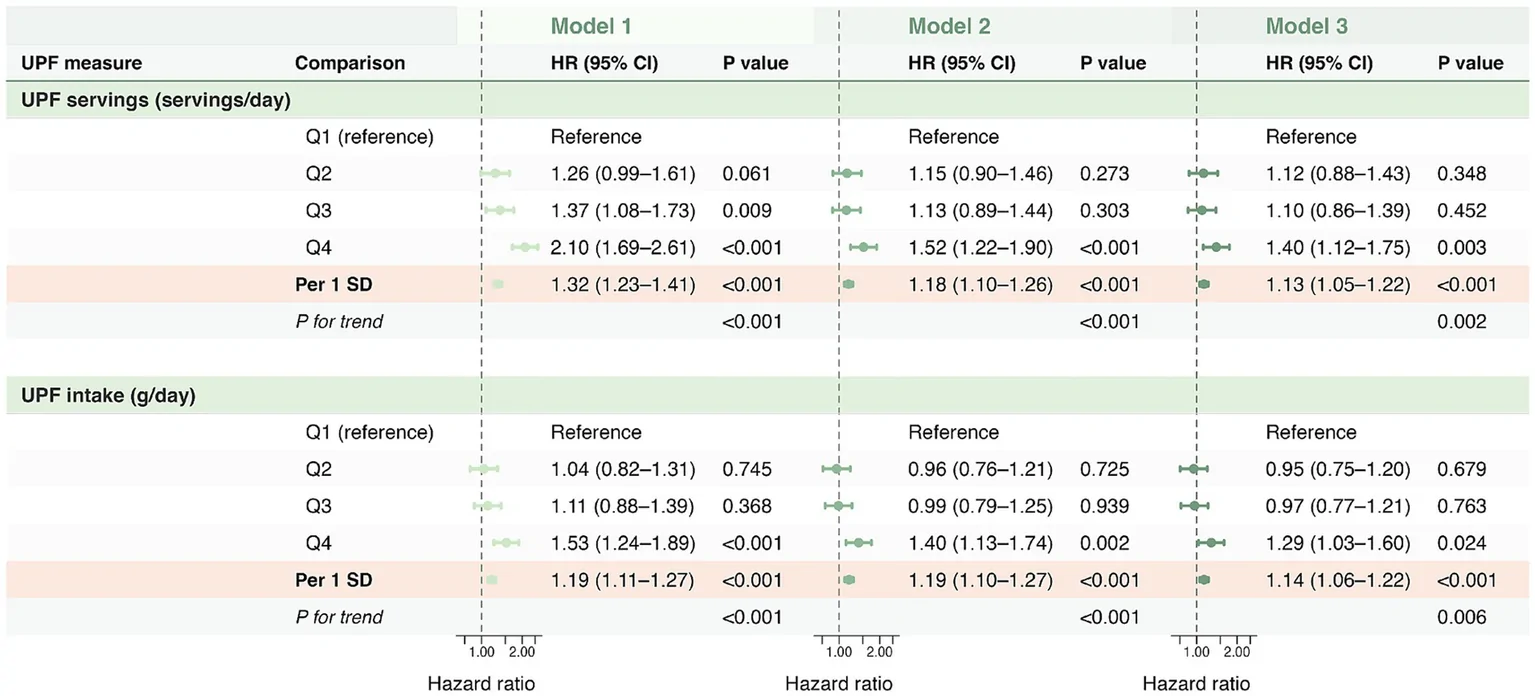
Hazard ratios for the association between ultra-processed food consumption and risk of atherosclerosis. Forest plot displaying HRs (95% CIs) for quartiles of UPF intake across three sequential models. Q1 (lowest intake) serves as the reference. Model 1 was unadjusted. Model 2 was adjusted for age, sex, ethnicity, education, Townsend deprivation index, smoking status, alcohol use, and IPAQ physical activity. Model 3 was adjusted for age, sex, ethnicity, education, Townsend deprivation index, smoking status, alcohol use, IPAQ physical activity, BMI, CRP and doctor-diagnosed diabetes. CI, confidence interval; HR, hazard ratio; SD, standard deviation.

**Figure 3:**
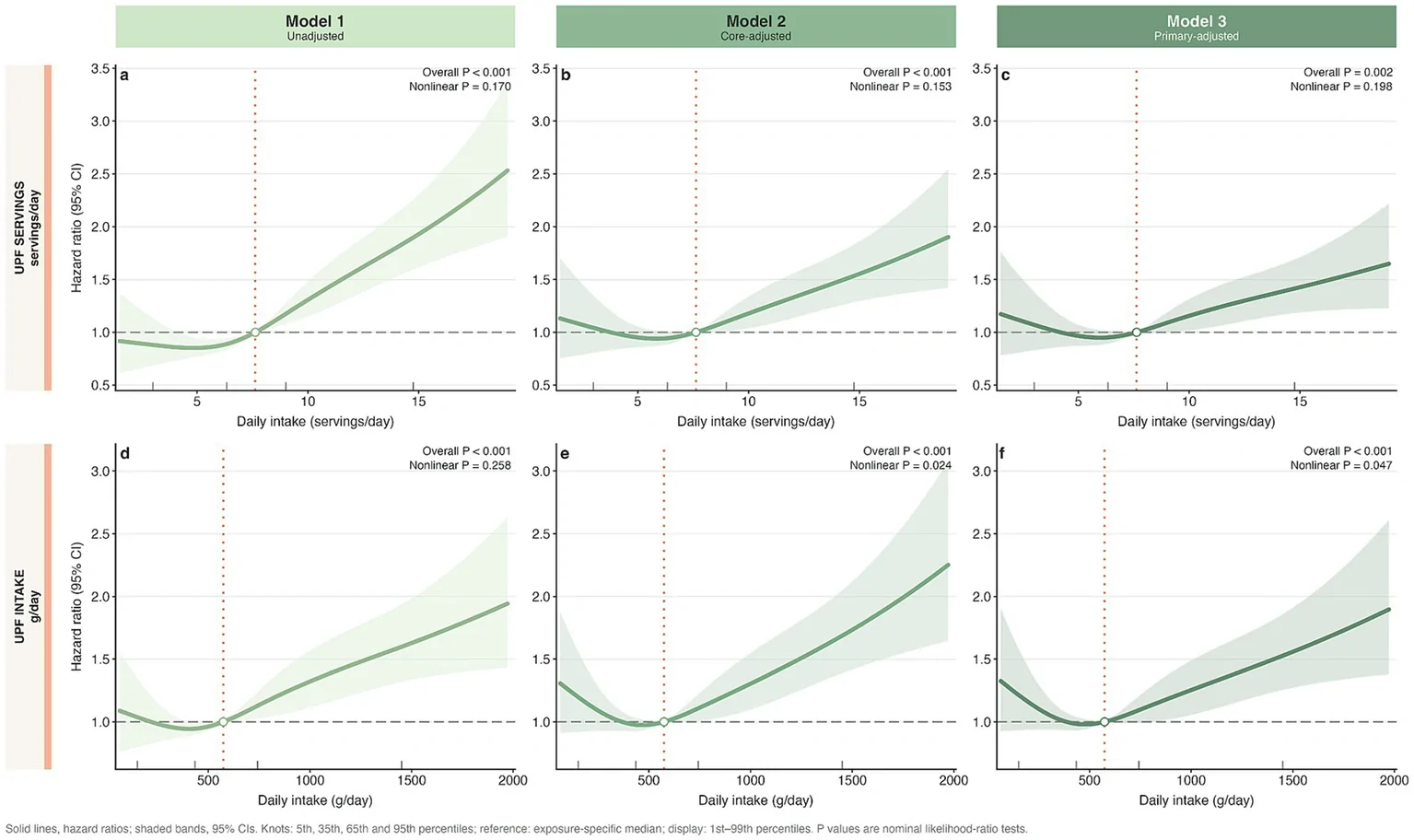
Panels **(a–c)** show UPF intake in servings/day for Models 1–3, respectively; panels **(d–f)** show UPF intake in g/day for Models 1–3, respectively. Hazard ratios were estimated using restricted cubic splines with knots at the 5th, 35th, 65th, and 95th percentiles, with the exposure-specific median as the reference value (*HR* = 1.00); estimates were displayed across the 1st–99th percentiles. The reference values were 7.63 servings/day and 573.83 g/day. Model 1 was unadjusted. Model 2 was adjusted for age, sex, ethnicity, education, Townsend deprivation index, smoking status, alcohol use, and IPAQ physical activity. Model 3 was adjusted for age, sex, ethnicity, education, Townsend deprivation index, smoking status, alcohol use, IPAQ physical activity, BMI, CRP and doctor-diagnosed diabetes.

Figure 2 illustrates the association between ultra-processed food (UPF) intake—measured in servings/day and grams/day—and incident clinically coded atherosclerosis (I70). In Model 3, a higher UPF intake was significantly associated with an increased risk of atherosclerosis. Participants in the highest quartile (Q4) of servings/day showed an elevated risk (HR 1.40, 95% CI 1.12–1.75; *p* = 0.003) compared to the lowest quartile (Q1). Similarly, Q4 of grams/day demonstrated a significantly increased risk (HR 1.29, 95% CI 1.03–1.60; *p* = 0.024). Per 1 SD increments also yielded consistent positive associations across all models (*p* < 0.001). Figure 3 presents restricted cubic spline analyses in the primary cohort. Restricted cubic spline analyses showed significant overall associations for both servings/day and grams/day. In Model 3, *P*_overall_ = 0.002 and *P*_nonlinearity_ = 0.198 for servings/day, whereas *P*_overall_ < 0.001 and *P*_nonlinearity_ = 0.047 for grams/day. These analyses characterize the shape of the associations and do not establish a clinical threshold. In Models 4–7, further covariate adjustment attenuated the associations; nevertheless, both measures of UPF intake remained positively associated with atherosclerosis risk in the step-specific available and common complete-case samples, although most 95% CIs included unity (Supplementary Figure S1).

### 3.3 Subgroup and sensitivity analyses

Model 3 subgroup analyses evaluating the association between ultra-processed food (UPF) intake and incident atherosclerosis are presented by Supplementary Figure S2. The positive associations per 1 SD increase in servings/day and grams/day remained largely consistent across various demographic, lifestyle, and clinical subgroups, including age, BMI, smoking status, and physical activity. Most interaction tests showed no significant effect modification (*P*_interaction_ > 0.05). Although the association appeared numerically different across KDM-age strata, the interaction analysis provided insufficient evidence of effect modification.

To assess robustness to reliance on a single recall, we repeated the analyses among participants with at least two valid WebQ assessments. Supplementary Figure S3 presents sensitivity analyses restricted to 87,924 participants with ≥2 WebQ assessments (385 events). UPF g/day showed significant associations using the subset-specific scale (Q4 vs. Q1: HR 1.35, 95% CI 1.01–1.81; *p* = 0.040) and per-SD analysis (*p* = 0.012). In contrast, UPF servings/day associations with incident atherosclerosis were positive but not statistically significant (Q4 vs. Q1: HR 1.30, 95% CI 0.96–1.77; *p* = 0.094; per SD: HR 1.10, 95% CI 0.99–1.22; *p* = 0.069). Overall, these sensitivity-analysis results were consistent with the main analysis.

### 3.4 Missing-data analyses

Among 172,329 eligible participants, 133,797 were included and 38,532 excluded (Supplementary Figure S4). Education showed the largest standardized difference (SMD = 0.246), followed by sex (SMD = 0.189). Exclusion was predominantly attributable to missing IPAQ activity (74.2%) and CRP (25.7%) data.

After multiple imputation, higher UPF intake remained associated with incident atherosclerosis (Supplementary Figure S5). In Model 3, the highest versus lowest quartile of UPF intake (servings/day) was associated with greater risk (HR 1.30, 95% CI 1.07–1.58, *p* = 0.008; *P*_trend_ = 0.007). The corresponding association for UPF intake (g/day) was weaker (HR 1.15, 95% CI 0.96–1.39, *p* = 0.132), although the trend remained significant (*P*_trend_ = 0.041). Each 1-SD increase in either measure was associated with 10% higher risk.

### 3.5 Identification of the metabolic signature and its associations with atherosclerosis

**Figure 4:**
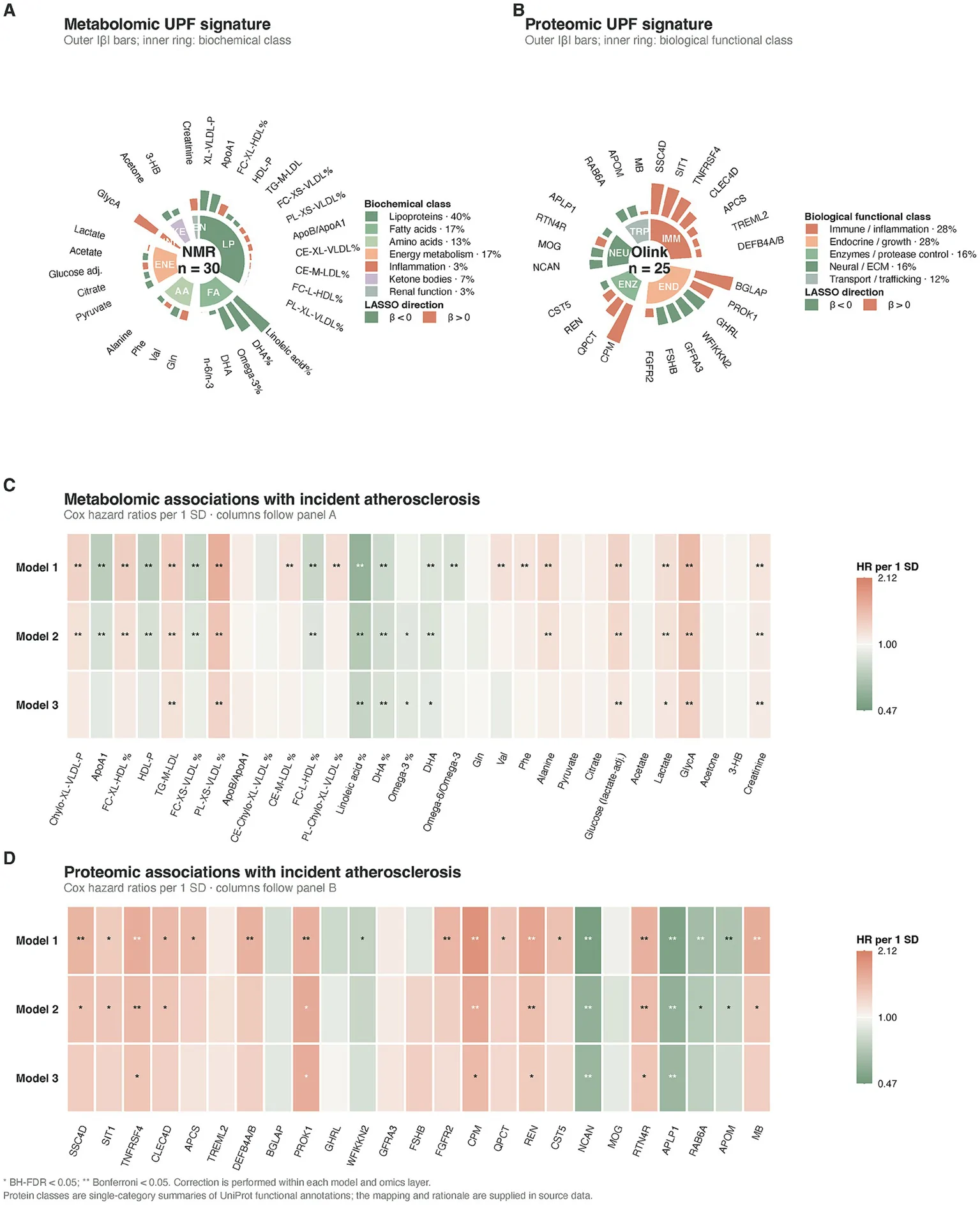
Identification and characterization of metabolomic and proteomic signature associated with atherosclerosis. **(A)** Metabolomic UPF signature. Sparse LASSO-selected metabolites contributing to the UPF-related metabolomic signature. The outer labels indicate individual biomarkers; inner-ring colors represent biochemical classes, and bar colors indicate the direction of the LASSO coefficient (β < 0, green; β > 0, orange). The center indicates the number of selected metabolites (*N* = 30). **(B)** Proteomic UPF signature. Sparse LASSO-selected proteins contributing to the UPF-related proteomic signature. The outer labels indicate individual proteins; inner-ring colors represent biological functional classes, and bar colors indicate the direction of the LASSO coefficient (β < 0, green; β > 0, orange). The center indicates the number of selected proteins (*N* = 25). **(C)** Metabolomic association s with incident atherosclerosis. Heatmap showing hazard ratios per 1-standard-deviation increase in individual metabolites across sequential adjustment models. Models 1–3 represent progressively adjusted Cox proportional hazards models. Color intensity indicates the magnitude and direction of association, with orange indicating higher and green indicating lower hazard ratios; white denotes estimates close to the null. Asterisks indicate statistical significance. **(D)** Proteomic associations with incident atherosclerosis. Heatmap showing hazard ratios per 1-standard-deviation increase in individual proteins across sequential adjustment models. Color intensity indicates the magnitude and direction of association, with orange indicating higher and green indicating lower hazard ratios; white denotes estimates close to the null. Asterisks indicate statistical significance. UPF, ultra-processed food; HR, hazard ratio; SD, standard deviation; LASSO, least absolute shrinkage and selection operator; NMR, nuclear magnetic resonance. *BH-FDR < 0.05; **Bonferroni < 0.05. Model 1 was unadjusted. Model 2 was adjusted for age, sex, ethnicity, education, Townsend deprivation index, smoking status, alcohol use, and IPAQ physical activity. Model 3 was adjusted for age, sex, ethnicity, education, Townsend deprivation index, smoking status, alcohol use, IPAQ physical activity, BMI, CRP, and doctor-diagnosed diabetes. LASSO coefficients represent predictive weights and should not be interpreted as causal effects.

UPF-related metabolomic signatures identified using sparse LASSO and their associations with incident atherosclerosis across sequentially adjusted Cox models are shown in Figure 4. Overall, the two signatures converged on lipid dysregulation, altered energy metabolism, systemic inflammation, and immune-endocrine or vascular-remodeling pathways.

The metabolomic signature comprised 30 NMR biomarkers, predominantly lipoproteins (40%), followed by fatty acids and glycolysis/energy-related metabolites (17% each), amino acids (13%), ketone bodies (7%), and inflammation- and renal/fluid-related markers (3% each) (Figure 4A). UPF intake was positively weighted for several glycolytic and lipoprotein measures, whereas inverse weights were observed mainly for fatty-acid, amino-acid, and selected lipoprotein traits. In Cox analyses, numerous metabolites were associated with atherosclerosis in Models 1 and 2. After multivariable adjustment (Model 3), higher TG-M-LDL, PL-XS-VLDL%, glucose, lactate, GlycA, and creatinine remained positively associated with risk, whereas linoleic acid%, DHA%, omega-3%, and selected lipid-related measures showed inverse associations (Figure 4C).

### 3.6 Identification of the proteomic signature and its associations with atherosclerosis

The proteomic signature included 25 proteins, distributed across immune/inflammatory (28%), endocrine/growth (28%), enzyme/protease-control (16%), neural/extracellular-matrix (16%), and transport/trafficking (12%) pathways (Figure 4B). Positive LASSO weights were observed for several inflammatory and endocrine proteins, including SSC4D, SIT1, TNFRSF4, CLEC4D, BGLAP, CPM, and MB, whereas proteins such as APLP1, RAB6A, APOM, and NCAN received inverse weights. In the Cox models, multiple proteins were associated with incident atherosclerosis before multivariable adjustment. In Model 3, TNFRSF4, PROK1, CPM, REN, RTN4R, and related proteins retained positive associations, while NCAN and APLP1 remained inversely associated after multiple-testing correction (Figure 4D).

## 4 Discussion

In this large-scale prospective cohort study of 133,797 UK Biobank participants, we identified a significant association between ultra-processed food (UPF) consumption and incident atherosclerosis risk. Participants in the highest quartile of UPF intake (g/day) demonstrated a 29% increased risk of atherosclerosis compared to those in the lowest quartile, with each standard deviation increment in UPF (g/day) consumption conferring a 14% elevated risk after multivariable adjustment. Through integrated metabolomic and proteomic profiling, we identified distinct molecular signatures characterized by dysregulated lipid metabolism, amino acid perturbations, chronic inflammation, and enzymatic alterations that were associated with UPF intake and subsequent atherosclerosis risk. These findings provide novel epidemiological evidence linking dietary patterns to atherosclerosis at both population and molecular levels, with important implications for preventive cardiology and public health policy.

Our findings extend previous research demonstrating adverse cardiovascular effects of UPF consumption. While the NutriNet-Santé cohort reported a 12% increase in cardiovascular disease risk per 10% increment in UPF contribution to total energy intake (5), and the Framingham Offspring Study documented significant associations with composite cardiovascular endpoints (19), these investigations primarily focused on cardiovascular events rather than the full pathological process of atherosclerosis. Previous studies have primarily examined composite cardiovascular events or mortality, whereas prospective evidence regarding clinically recorded atherosclerosis coded as ICD-10 I70 remains limited. This endpoint captures recognized clinical disease but does not measure subclinical plaque burden or the full pathological process of atherosclerosis. Our study addresses this gap by specifically examining atherosclerosis as the outcome, with an observed hazard ratio of 1.29 for the highest versus lowest quartile—clinically meaningful and comparable to established cardiovascular risk factors (28, 29). Nevertheless, given that UPF consumption frequently exceeds 60% of total energy intake in Western populations (7), even modest risk elevations translate into substantial population-attributable disease burden.

Our exploratory multi-omics analyses highlighted several overarching biological themes before consideration of individual biomarkers. At the metabolomic level, the dominant patterns involved atherogenic lipoprotein remodeling, altered glucose and energy metabolism, systemic inflammation, and lower relative levels of unsaturated fatty acids. At the proteomic level, the main themes were immune-inflammatory activation, endocrine and vascular signaling, proteolytic regulation, and extracellular-matrix or neural pathways. Together, these findings suggest that UPF consumption is associated with coordinated molecular perturbations potentially relevant to atherosclerosis, rather than changes in a single isolated pathway. Importantly, LASSO weights represent each biomarker’s contribution to the multivariable UPF-related signature and should not be interpreted as independent or causal effects.

The metabolomic signature comprised 30 NMR biomarkers and was dominated by lipoprotein measures (40%), followed by fatty-acid and glycolysis/energy-related metabolites (17% each). Within the first dominant theme—lipoprotein remodeling—higher TG-M-LDL and PL-XS-VLDL% remained associated with greater risk of clinically coded atherosclerosis after multivariable adjustment. These findings are consistent with the role of triglyceride-rich lipoproteins and their remnants in arterial lipid deposition, endothelial dysfunction, and vascular inflammation. UPFs commonly contain refined carbohydrates and unfavorable fat profiles, which may promote hepatic lipogenesis, triglyceride production, and remodeling of circulating lipoproteins. However, our observational findings do not establish that these lipid traits mediate the association between UPF intake and atherosclerosis.

The second metabolomic theme involved altered glucose and energy metabolism. Higher glucose and lactate remained positively associated with incident atherosclerosis after multivariable adjustment. Diets rich in rapidly absorbable carbohydrates may contribute to repeated glycemic excursions, insulin resistance, and increased glycolytic flux. Elevated lactate may also indicate metabolic inflexibility or altered oxidative metabolism, both of which have been linked to endothelial dysfunction and vascular disease. These findings suggest that disturbances in glucose utilization and energy metabolism may accompany the association between UPF consumption and atherosclerosis.

The third theme was systemic inflammation and related metabolic dysfunction. GlycA represents N-acetyl side chains of circulating acute-phase glycoproteins and has been consistently linked to cardiovascular events (30, 31), reflecting chronic low-grade inflammation—a fundamental driver of atherogenesis through endothelial activation, foam cell formation, and plaque instability (32). The positive association with creatinine may additionally reflect renal or broader systemic metabolic dysfunction. Nevertheless, these biomarkers were identified and evaluated in the same analytic sample, and their biological or prognostic interpretation therefore requires caution.

In contrast, the fatty-acid pattern was characterized by inverse associations of linoleic acid%, DHA%, and omega-3% with incident atherosclerosis. Docosahexaenoic acid (DHA), an omega-3 polyunsaturated fatty acid, demonstrated the strongest negative coefficient. DHA exerts anti-atherogenic effects through multiple pathways, including resolution of inflammation and improvement of endothelial function (33, 34). The inverse association likely reflects dietary displacement—diets high in UPFs are characteristically low in whole foods rich in omega-3 fatty acids (35). However, fatty-acid percentages are compositional measures; changes in one component necessarily correspond to changes in others and should not be interpreted as isolated nutrient effects.

The proteomic signature comprised 25 proteins, predominantly belonging to immune/inflammatory and endocrine/growth pathways (28% each), with additional representation of enzyme/protease-control, neural/extracellular-matrix, and transport/trafficking pathways. Positive LASSO weights for SSC4D, SIT1, TNFRSF4, CLEC4D, CPM, and MB indicate that these proteins contributed to the multivariable UPF-related pattern. As with the metabolomic signature, these weights reflect penalized feature selection rather than independent associations.

The principal proteomic theme was immune-inflammatory and vascular signaling. After multivariable adjustment and multiple-testing correction, TNFRSF4, PROK1, CPM, REN, and RTN4R, among other proteins, remained positively associated with incident atherosclerosis. TNFRSF4 (OX40) participates in T-cell co-stimulation and may contribute to sustained vascular immune activation (36). CPM is involved in peptide processing and macrophage-related inflammatory responses (37), whereas renin is central to the renin–angiotensin system, which regulates blood pressure, endothelial function, oxidative stress, and vascular remodeling (38). PROK1 and RTN4R may reflect broader angiogenic, inflammatory, neural, or tissue-remodeling processes (39, 40). Collectively, these findings point toward immune-endocrine and vascular pathways, although the context-dependent functions of circulating proteins preclude mechanistic inference.

A secondary proteomic theme involved extracellular-matrix and neural or cell-surface processes. NCAN and APLP1 remained inversely associated with atherosclerosis after multivariable adjustment and multiple-testing correction. NCAN is an extracellular-matrix proteoglycan (41), whereas APLP1 participates in neuronal and cell-surface processes (42). Their relevance to atherosclerosis is less established than that of conventional inflammatory and lipid markers. These inverse associations may reflect previously unrecognized pathways, correlated molecular profiles, or residual confounding; they should therefore be regarded as hypothesis-generating rather than evidence of protective effects.

Overall, the metabolomic and proteomic signatures converged on several biological themes: atherogenic lipoprotein remodeling, disrupted glucose and energy metabolism, altered fatty-acid composition, chronic inflammation, and immune-endocrine and vascular signaling. Longitudinal studies with repeated dietary and omics measurements, formal assessment of mediator–outcome temporality, and independent replication are needed to clarify these pathways.

Our findings support public-health recommendations emphasizing minimally processed foods and limiting UPF consumption. Practical approaches may include preparing meals from basic ingredients, selecting products with simpler ingredient lists, and replacing UPFs with fruits, vegetables, whole grains, legumes, nuts, and sources of unsaturated fatty acids. Population-level measures could include clear front-of-package labeling, restrictions on marketing to children, fiscal policies, and improved access to affordable minimally processed foods (43, 44). The effectiveness and equity implications of these interventions require direct evaluation.

### 4.1 Strengths and limitations

This investigation possesses several notable strengths, including large sample size with prospective design, standardized dietary assessment using the NOVA classification system, integration of high-throughput metabolomic and proteomic profiling, adjustment for measured confounders, and sensitivity analyses demonstrating consistency of findings.

Several limitations merit consideration. First, dietary assessment relied on self-reported 24-h recalls over a relatively short period. These assessments are susceptible to recall error and within-person variation and may not capture long-term changes in dietary behavior during follow-up, potentially causing exposure misclassification and attenuating the observed associations. Second, the UK Biobank cohort comprises predominantly White, middle-aged, relatively healthy individuals, potentially limiting generalizability. Third, residual and time-varying confounding remains possible, and adjustment for potential intermediates may attenuate the total association. Finally, the omics analyses were exploratory; therefore, the identified signatures require cautious interpretation and external replication.

## 5 Conclusion

This large-scale prospective cohort study provides evidence that higher UPF consumption is associated with increased risk of clinically coded atherosclerosis after multivariable adjustment. Integrated metabolomic and proteomic profiling revealed distinct molecular signatures characterized by systemic inflammation, lipid dysregulation, amino acid perturbations, and enzymatic alterations. These findings support dietary recommendations emphasizing whole, minimally processed foods for cardiovascular disease prevention and highlight candidate molecular correlates requiring independent validation. Given the high prevalence of UPF consumption in contemporary diets and the substantial burden of atherosclerotic cardiovascular disease, our results underscore the public health imperative for policies and interventions aimed at transforming food environments toward healthier dietary patterns.

## Data Availability

The data analyzed in this study are subject to the following licenses/restrictions: The data used in this study were available from the UK Biobank with restrictions applied. The data were used under license. The UK Biobank Resource holds the data used in this article. Data can be accessed by application to the UK Biobank (https://www.ukbiobank.ac.uk/). Requests to access these datasets should be directed to https://www.ukbiobank.ac.uk/.
