# Supplementary material for: Ultra-processed food consumption and atherosclerosis risk: a prospective cohort study of UK Biobank
Source: Front Nutr. 2026 Sep 10;13:1925613. doi: 10.3389/fnut.2026.1925613 (PMC13600853; doi:10.3389/fnut.2026.1925613)
Supplement: Supplementary file 1 [file Supplementary_file_1.DOCX]

Supplementary Material

# 1 Supplementary Tables

Table S1. Data sources and participant numbers at each screening stage for atherosclerosis in the UK Biobank.

| Population order | Population id | Population label | Family order | Source family | count | denominator |
| --- | --- | --- | --- | --- | --- | --- |
| 1 | Raw first report | All participants with an I70 first-reported date | 1 | Death register | 213 | 4823 |
| 1 | Raw first report | All participants with an I70 first-reported date | 2 | Primary care | 160 | 4823 |
| 1 | Raw first report | All participants with an I70 first-reported date | 3 | Hospital admissions | 4450 | 4823 |
| 1 | Raw first report | All participants with an I70 first-reported date | 4 | Self-report | 0 | 4823 |
| 2 | Incident event | Incident I70 events after the diet index date | 1 | Death register | 24 | 877 |
| 2 | Incident event | Incident I70 events after the diet index date | 2 | Primary care | 12 | 877 |
| 2 | Incident event | Incident I70 events after the diet index date | 3 | Hospital admissions | 841 | 877 |
| 2 | Incident event | Incident I70 events after the diet index date | 4 | Self-report | 0 | 877 |
| 3 | Fixed model 3 event | Incident I70 events in the locked Model 3 cohort | 1 | Death register | 20 | 654 |
| 3 | Fixed model 3 event | Incident I70 events in the locked Model 3 cohort | 2 | Primary care | 9 | 654 |
| 3 | Fixed model 3 event | Incident I70 events in the locked Model 3 cohort | 3 | Hospital admissions | 625 | 654 |
| 3 | Fixed model 3 event | Incident I70 events in the locked Model 3 cohort | 4 | Self-report | 0 | 654 |

Table S2. Baseline characteristics of the participants (UPF: servings/day).

| Characteristics | Q1 | Q2 | Q3 | Q4 | *p* |
| --- | --- | --- | --- | --- | --- |
|  | (n=35,692) | (n=31,481) | (n=33,360) | (n=33,264) |  |
| UPF servings/day (median [IQR]) | 4.25 [3.17–5.00] | 6.62 [6.00–7.00] | 8.83 [8.17–9.50] | 12.25 [11.00–14.00] |  |
| Age at diet baseline, years, Mean (SD) | 58.2 (7.8) | 58.7 (7.8) | 58.8 (8.0) | 58.9 (8.1) | <0.001 |
| Race or ethnicity (%) |  |  |  |  | <0.001 |
| White | 33,811 (94.7) | 30,553 (97.1) | 32,656 (97.9) | 32,692 (98.3) |  |
| Non-White | 1,881 (5.3) | 928 (2.9) | 704 (2.1) | 572 (1.7) |  |
| Education (%) |  |  |  |  | <0.001 |
| college | 19,505 (54.6) | 15,658 (49.7) | 15,241 (45.7) | 13,076 (39.3) |  |
| high school | 14,565 (40.8) | 14,134 (44.9) | 16,004 (48.0) | 17,487 (52.6) |  |
| less than high school | 1,622 (4.5) | 1,689 (5.4) | 2,115 (6.3) | 2,701 (8.1) |  |
| Sex (%) |  |  |  |  | <0.001 |
| Female | 22,962 (64.3) | 18,360 (58.3) | 17,023 (51.0) | 12,939 (38.9) |  |
| Male | 12,730 (35.7) | 13,121 (41.7) | 16,337 (49.0) | 20,325 (61.1) |  |
| Alcohol drinking status (%) |  |  |  |  | <0.001 |
| Never | 988 (2.8) | 772 (2.5) | 858 (2.6) | 952 (2.9) |  |
| Previous | 929 (2.6) | 764 (2.4) | 871 (2.6) | 1,109 (3.3) |  |
| Current | 33,775 (94.6) | 29,945 (95.1) | 31,631 (94.8) | 31,203 (93.8) |  |
| Ever smoked (%) |  |  |  |  | <0.001 |
| No | 14,742 (41.3) | 12,910 (41.0) | 13,049 (39.1) | 12,284 (36.9) |  |
| Yes | 20,950 (58.7) | 18,571 (59.0) | 20,311 (60.9) | 20,980 (63.1) |  |
| CRP group (%) |  |  |  |  | <0.001 |
| Below median | 19,524 (54.7) | 16,445 (52.2) | 16,789 (50.3) | 15,496 (46.6) |  |
| At or above median | 16,168 (45.3) | 15,036 (47.8) | 16,571 (49.7) | 17,768 (53.4) |  |
| BMI, kg/m2, (%) |  |  |  |  | <0.001 |
| < 30 | 30,155 (84.5) | 26,084 (82.9) | 26,774 (80.3) | 24,933 (75.0) |  |
| ≥30 | 5,537 (15.5) | 5,397 (17.1) | 6,586 (19.7) | 8,331 (25.0) |  |
| Doctor-diagnosed diabetes (%) |  |  |  |  | <0.001 |
| No | 34,808 (97.5) | 30,507 (96.9) | 32,117 (96.3) | 31,416 (94.4) |  |
| Yes | 884 (2.5) | 974 (3.1) | 1,243 (3.7) | 1,848 (5.6) |  |
| Townsend deprivation index, Mean (SD) | -1.42 (2.98) | -1.75 (2.79) | -1.87 (2.73) | -1.77 (2.80) | <0.001 |
| Physical activity (IPAQ) (%) |  |  |  |  | <0.001 |
| Low | 5,952 (16.7) | 5,571 (17.7) | 6,219 (18.6) | 6,628 (19.9) |  |
| Moderate | 15,161 (42.5) | 13,729 (43.6) | 14,487 (43.4) | 13,932 (41.9) |  |
| High | 14,579 (40.8) | 12,181 (38.7) | 12,654 (37.9) | 12,704 (38.2) |  |

## Characteristics of participants are presented as mean (SD) for continuous variables, and frequency (%) for categorical variables.

Abbreviation: UPF, ultra-processed food; CRP, C-reactive protein; BMI, body mass index.

# 2 Supplementary Figures

**Figure S1. Associations of ultra-processed food intake with incident atherosclerosis across sequentially adjusted models using step-specific available and common complete-case samples.**

Model 2 was adjusted for age, sex, ethnicity, education, Townsend deprivation index, smoking status, alcohol use, and IPAQ physical activity. Model 3 was adjusted for age, sex, ethnicity, education, Townsend deprivation index, smoking status, alcohol use, IPAQ physical activity, BMI, CRP and doctor-diagnosed diabetes. Model 4 further adjusted for the DASH diet score. Model 5 additionally adjusted for total energy intake. Model 6 included systolic blood pressure and LDL-C as additional covariates. Model 7 considered the use of lipid-lowering or antihypertensive medications.

Abbreviation: UPF, ultra-processed food.

**Figure S2. Subgroup analyses of the associations between ultra-processed food intake and incident atherosclerosis.**

Forest plot showing hazard ratios (95% CIs) for the association between UPF consumption with incident atherosclerosis across prespecified subgroups.

Abbreviation: UPF, ultra-processed food; CI, confidence interval; HR, hazard ratio; SD, standard deviation.

**Figure S3. Sensitivity analyses using repeated dietary assessments of ultra-processed food intake and incident atherosclerosis.**

Forest plots show hazard ratios (HRs) and 95% confidence intervals (CIs) for the associations between ultra-processed food (UPF) intake and incident atherosclerosis across predefined sensitivity analyses based on repeated Oxford WebQ dietary assessments. Associations are presented for the highest versus lowest quartile (Q4 vs Q1) and per 1-standard-deviation (SD) increase in UPF intake, quantified as servings per day and grams per day.

Abbreviation: UPF, ultra-processed food; CI, confidence interval; HR, hazard ratio; SD, standard deviation; WebQ, Oxford WebQ dietary assessment.

**Figure S4. Characteristics and covariate missingness among participants included in and excluded from the primary Model 3 analysis**

A, Absolute standardized mean differences (SMDs) in non-missing baseline characteristics between included and excluded participants; the dashed line indicates ∣SMD∣= 0.10. B, Prevalence of missing Model 3 covariates among excluded participants. Participants could have multiple missing covariates.

Abbreviation: UPF, ultra-processed food; CRP, C-reactive protein; BMI, body mass index; WebQ, Oxford WebQ dietary assessment.

**Figure S5. Associations of ultra-processed food intake with incident atherosclerosis: a multiple-imputation sensitivity analysis**

Model 1 was unadjusted. Model 2 was adjusted for age, sex, ethnicity, education, Townsend deprivation index, smoking status, alcohol use, and IPAQ physical activity. Model 3 was adjusted for age, sex, ethnicity, education, Townsend deprivation index, smoking status, alcohol use, IPAQ physical activity, BMI, CRP and doctor-diagnosed diabetes.

Abbreviation: UPF, ultra-processed food; CI, confidence interval; HR, hazard ratio; SD, standard deviation.
